# The aryl hydrocarbon receptor maintains antitumor activity of liver resident natural killer cells after partial hepatectomy in C57BL/6J mice

**DOI:** 10.1002/cam4.6554

**Published:** 2023-09-25

**Authors:** Koki Sato, Masahiro Ohira, Yuki Imaoka, Kouki Imaoka, Tomoaki Bekki, Marlen Doskali, Ryosuke Nakano, Takuya Yano, Yuka Tanaka, Hideki Ohdan

**Affiliations:** ^1^ Department of Gastroenterological and Transplant Surgery, Graduate School of Biomedical and Health Sciences Hiroshima University Hiroshima Japan; ^2^ Medical Center for Translational and Clinical Research Hiroshima University Hospital Hiroshima Japan

**Keywords:** antitumor activity, cancer, immunology, liver immune system, natural killer cells

## Abstract

**Background:**

Liver‐resident natural killer (lr‐NK) cells are distinct from conventional NK cells and exhibit higher cytotoxicity against hepatoma via tumor necrosis factor‐related apoptosis‐inducing ligand (TRAIL). However, the mechanism by which partial hepatectomy (PH) significantly suppresses TRAIL expression in lr‐NK cells remains unclear.

**Methods:**

This study aimed to investigate the PH influence on the function and characteristics of liver‐resident NK (lr‐NK) cells using a PH mouse model.

**Results:**

Here, we report that PH alters the differentiation pattern of NK cells in the liver, and an aryl hydrocarbon receptor (AhR) molecule is involved in these changes. Treatment with the AhR agonist 6‐formylindolo[3,2‐b]carbazole (FICZ) inhibited the maturation of NK cells. FICZ increased the immature subtype proportion of NK cells with high TRAIL activity and decreased the mature subtype of NK cells with low TRAIL activity. Consequently, FICZ increased the expression of TRAIL and cytotoxic activity of NK cells in the liver, and this effect was confirmed even after hepatectomy. The participation of AhR promoted FoxO1 expression in the mTOR signaling pathway involved in the maturation of NK cells, resulting in *TRAIL* expression.

**Conclusion:**

Our findings provide direct in‐vivo evidence that partial hepatectomy affects lrNK cell activity through NK cell differentiation in the liver. Perioperative therapies using an AhR agonist to improve NK cell function may reduce the recurrence of hepatocellular carcinoma after hepatectomy.

## 1 INTRODUCTION

Natural killer (NK) cells play a crucial role in the innate immune response against tumors and virus‐infected cells.^1^ Their activity is regulated by various activating and inhibitory receptors.^2^ The liver contains abundant NK cells, termed “liver‐resident NK cells” (lr‐NK), in both humans and mice. Lr‐NK cells have higher cytotoxicity through tumor necrosis factor‐related apoptosis‐inducing ligand (TRAIL) than NK cells from the spleen and peripheral blood^3^, ^4^, ^5^ and are characterized by eomesodermin (eomes)^−^ DX5^−^ TRAIL^+^ T‐bet^+^ as an immature population.^6^, ^7^, ^8^ NK cells are a heterogeneous population, and their maturation can be classified based on the surface expression of CD27 and CD11b. CD11b^low^ CD27^low^, CD11b^low^ CD27^high^, CD11b^high^ CD27^high^, and CD11b^high^ CD27^low^ (hereafter, double negative: DN, CD27 single positive: CD 27 SP, double positive: DP, and CD11b single positive: CD11b SP NK cells, respectively) are proposed as the discrete stages of in‐vivo maturation that follow a pathway from the DN (immature) to the CD11b SP (mature) stage.^9^ We have previously reported that immature CD27 SP NK cells have high TRAIL expression in the liver.^10^ Clinically, TRAIL expression in lr‐NK cells is significantly decreased after partial hepatectomy (PH).^4^, ^11^ Consequently, reduced antitumor activity causes postoperative tumor recurrence.^4^ However, the mechanism by which TRAIL expression decreases after PH has not been elucidated.

Aryl hydrocarbon receptors (AhRs) are transcription factors belonging to the basic helix–helix family that activates various ligands. They were originally discovered as a P450 pathway induction factor in response to environmental contamination, such as 2,3,7,8‐tetrachlorodibenzo‐*p*‐dioxin (TCDD).^12^ Exposure to TCDD produces tissue‐specific toxic and biological effects, the majority being AhR‐dependent.^12^ AhR was first reported to bind the xenobiotic compound dioxin; however, it is now known to bind a wide range of natural exogenous and endogenous ligands,^12^ such as TCDD, FICZ, kynurenine, or 2‐(1′H‐indole‐3′‐carbonyl)‐thiazole‐4‐carboxylic acid methyl ester (ITE). In particular, AhR expression is highly prevalent in DX5‐ lr‐NK cells^13^ and it is critical for the maintenance of lr‐NK cells in mouse models.^14^ During normal NK cell development, AhR activation hinders the transition from the third to the fourth maturation stage, while AhR antagonists facilitate differentiation to the fourth stage.^15^, ^16^ Based on these observations, we hypothesized that AhR plays an important role in maintaining the antitumor activity of lr‐NK cells following PH. To investigate this, our study aimed to analyze the mechanisms involved in the differentiation of lr‐NK cells and expression of TRAIL using 6‐formylindolo[3,2‐b]carbazole (FICZ), a ligand of AhR. Furthermore, the effect of FICZ on lr‐NK cells after PH and its antitumor activity was investigated in a mouse hepatectomy model.

## 2 MATERIALS AND METHODS

### 2.1 Mice

Female mice C57BL/6J (B6) aged 8–12 weeks were obtained from CLEA Japan, Inc. and housed in a pathogen‐free microenvironment within the animal facility at Hiroshima University, Japan. All animal procedures were conducted according to the principles outlined in the Guide for the Care and Use of Laboratory Animals and were approved by the Ethics Review Committee for Animal Experimentation of the Graduate School of Biomedical Sciences, Hiroshima University (approval number: A20‐97).

### 2.2 Partial hepatectomy of mice

The mice were anesthetized with isoflurane, and the large median lobe and left lateral lobe of their livers were carefully ligated and excised. Approximately 70% of the total liver mass was removed by resecting portions of the hepatic parenchyma. Post‐surgery, the mice were placed on a heating pad to facilitate their recovery. PH was performed as described by Yano et al.^11^

### 2.3 Establishment of animal model

Mice were randomly and blindly divided into two groups: sham (control) and AhR ligand FICZ, an endogenous ligand derived from tryptophan (FICZ), groups. Mice received three intravenous injections of 3 mg of FICZ for 1 week before PH. The AhR antagonist, CH223191, was injected intraperitoneally at a single dose of 1 mg/kg of body weight three times for 1 week, based on a previous study.^17^

### 2.4 Isolation of lymphocytes

To isolate liver lymphocytes, we followed a previously established protocol.^11^ Briefly, 1 mL phosphate‐buffered saline (PBS) supplemented with 10% heparin was injected through the portal vein. The liver was dissected and perfused with 50 mL PBS containing 0.1% ethylenediaminetetraacetic acid. Blood cells from the liver perfusate were collected through centrifugation, and erythrocytes were eliminated using ammonium‐chloride‐potassium (ACK) lysis buffer.

### 2.5 Flow cytometry

Flow cytometric analysis was performed using a FACS Canto II cytometer (BD Biosciences). Freshly isolated mononuclear cells were preincubated with anti‐CD16/32 (2.4G2) monoclonal antibody (mAb) to prevent nonspecific binding of the Fcγ II/III receptor. Subsequently, the cells were stained with appropriately diluted fluorescently labeled mAbs. For phenotyping of NK cell surface markers, liver and spleen leukocytes were stained with the following mAbs: anti‐NK1.1 (PK136), anti‐CD69 (H1.2F3), anti‐Dx5 (CD49b; all from BD Pharmingen) and anti‐TCRβ chain and anti‐TRAIL (CD253; BioLegend). Dead cells were excluded from the analysis through forward‐scatter and propidium iodide (PI; Sigma‐Aldrich) or 7‐amino‐actinomycin D (7‐AAD; BD Biosciences) staining.

### 2.6 Quantitative RT‐PCR

Total RNA was isolated from the mouse samples using an RNeasy Mini Kit (Qiagen), and reverse‐transcribed using a QuantiTect reverse transcript kit (Qiagen) according to the manufacturer's protocol. Quantitative RT‐PCR was conducted in triplicate with a reaction volume of 20 μL, using the SYBR Green PCR Master Mix (Applied Biosystems) as per the manufacturer's instructions. Gene expression was assessed using the Rotor‐GeneQ instrument (Qiagen). The amplification protocol comprised an initial denaturation step at 95°C for 5 min, followed by 40 cycles of denaturation at 95°C for 5 s and annealing/extension at 60°C for 10 s. Relative gene expression was determined using the ΔΔCt method and normalized to the expression of β‐2 microglobulin as a reference gene. The PCR primer sequences utilized in this study are listed in Table S1.

### 2.7 Cytotoxicity assay

Cell cytotoxicity was evaluated as previously described.^4^, ^18^ Briefly, Hepa1‐6 cells were labeled with Na2[^51^Cr]O4 and used as target cells. Subsequently, the target cells were incubated with effector cells in round‐bottomed 96‐well plates at 37°C for 4 h. The percentage of cytotoxicity, determined by measuring ^51^Cr release, was calculated using the following formula: percent cytotoxicity = [(cpm of experimental release − cpm of spontaneous release)]/[(cpm of maximum release − cpm of spontaneous release)] × 100. All cytotoxicity assays were performed in triplicate to ensure the reliability of the results.

### 2.8 Histological evaluation of metastatic growth in the liver

To induce tumors in vivo, Hepa1‐6 cells were injected into the spleen using a medium containing 0.2 mL medium 199 (Sigma‐Aldrich) with a concentration of 10^7^ cells/mL.^18^ The injection was carried out slowly after identifying the spleen. After 7 days of tumor cell injection, the mice were sacrificed, and the liver was extracted and fixed in 10% formalin overnight. Tissue sections of the liver (4 μm thickness) were stained with hematoxylin and eosin (HE) for further analysis. The relative areas occupied by the tumors were calculated as a percentage of the total scanned liver area using a BZ‐8000 microscope (Keyence).

### 2.9 Microarray analysis

The Agilent RNA 6000 Pico Kit (Agilent Technologies) was used to measure RNA quality using an Agilent 2100 Bioanalyzer. A GeneChip™ WT PLUS Reagent Kit (Thermo Fisher) was used to process 100 ng of total RNA into cRNA, which was further processed into biotin‐labeled single‐stranded DNA. Single‐strand DNA (2.3 μg) was hybridized with the Clariom S Array (Thermo Fisher) in a GeneChip® Hybridization Oven 645 (Affymeterix) using a GeneChip™ Hybridization, Wash, and Stain Kit (Thermo Fisher) and a GeneChip® Fluidics Station 450 (Affymetrix). The array was scanned using a GeneChip® Scanner 3000 7G (Affymetrix). A CEL file was automatically created after scanning and analyzed using Transcriptome Analysis Console (TAC) ver. 4.0. Lastly, a text file and a figure of the expression level were generated.

### 2.10 GeneSpring GX

The raw data in the .CEL format was subjected to analysis using the GeneSpring software (v14.9.1, Agilent). To ensure accurate comparisons, the samples were normalized and transformed using the Robust Multi‐Array Analysis method. The gene‐level analysis was conducted by averaging the expression values of all the probes corresponding to the same probe ID. Probes that did not exhibit >2‐fold change were excluded from the analysis. Next, significantly regulated genes were extracted and exported to Excel files for subsequent downstream analysis.

### 2.11 Ingenuity pathway analysis

The list of significant gene entities was uploaded to the Ingenuity Pathway Analysis software (IPA, Qiagen). The software's “core analysis” feature was employed to analyze the differentially expressed genes, exploring various aspects such as biological processes, canonical pathways, upstream transcriptional regulators, and gene networks. Each gene identifier was mapped to its respective gene object within the Ingenuity Pathway Knowledge Base to facilitate comprehensive analysis and interpretation.

### 2.12 Statistical analysis

Independent sample *t*‐tests and nonparametric Mann–Whitney *U*‐tests were performed to determine the significance of differences between the two independent groups. ANOVA was used to evaluate the statistical significance of variations in cytotoxicity among the groups. Results with *p* < 0.05 were considered statistically significant. All data are presented as the mean ± standard deviation (SD). Statistical analyses were performed using the JMP 14 software (SAS Institute Inc.).

## 3 RESULTS

### 3.1 NK cell differentiation is altered following partial hepatectomy

**FIGURE 1:**
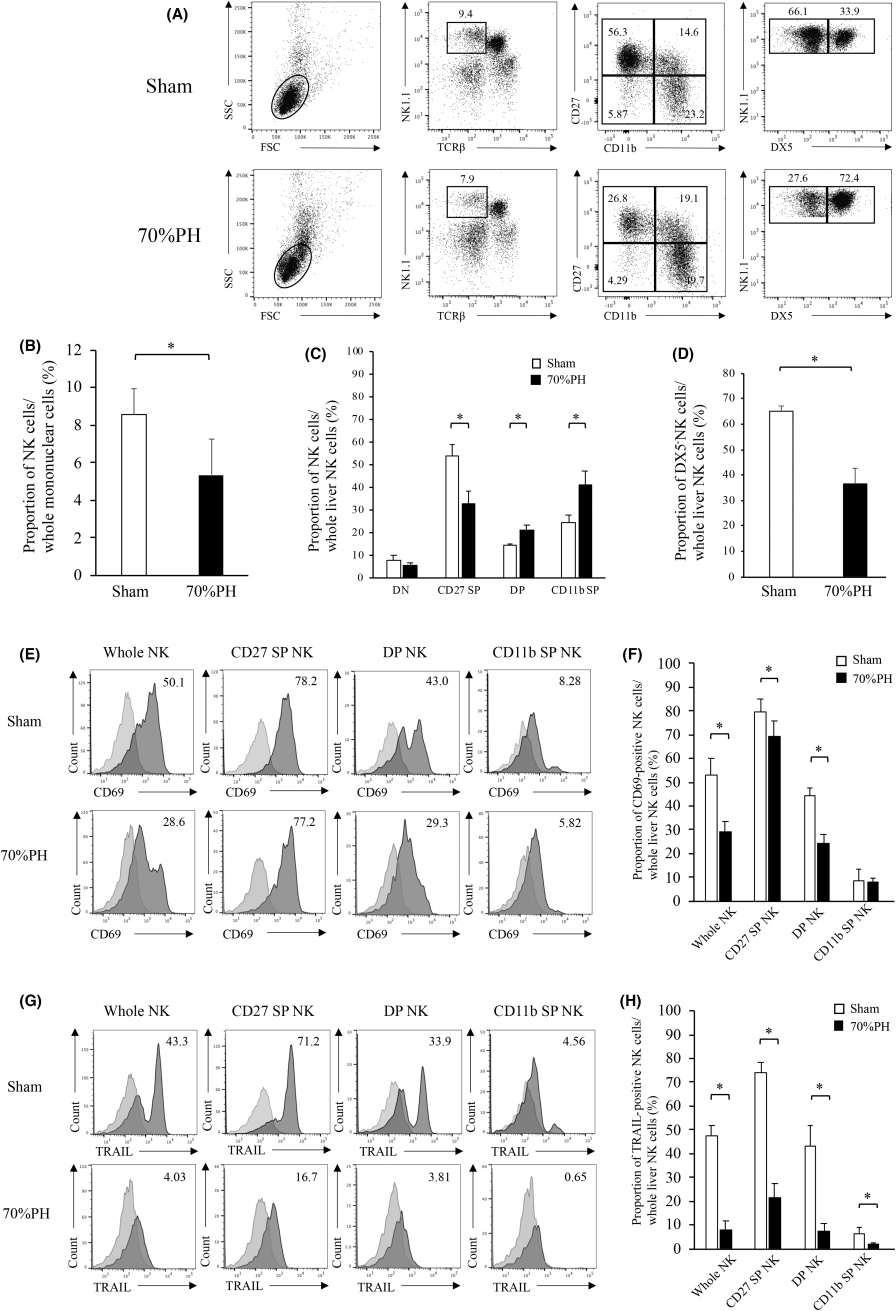
NK Cell differentiation is altered following partial hepatectomy. (A) Samples are shown from the sham (upper) and PH groups 3 days post operation after 70% PH (lower). (B) Proportions of identified NK1.1^+^TCRβ^−^ lr‐NK cells are shown (sham *n* = 5, PH *n* = 5). (C) Proportion of lr‐NK cell subsets in sham and PH‐treated mice. (D) The proportion of DX5^−^ and DX5^+^ NK cells in sham and PH‐treated mice. (E) Representative histograms for CD69 expression in each subset are shown for sham (upper) and PH‐treated (lower) mice. (F) The bar graph shows the average expression of CD69‐positive NK cells in each subset for sham and PH‐treated mice. (G) Representative histograms for TRAIL expression in each subset are shown for sham (upper) and PH‐treated (lower) mice. (H) The bar graph shows the average expression for TRAIL‐positive NK cells of each subset in sham or PH mice. Independent samples *t*‐tests were used to assess the statistical significance of differences between groups, when appropriate. *p < 0.05.

To assess the influence of PH on the maturation of lr‐NK cells, we first analyzed the phenotype of lr‐NK cells obtained from mice who underwent PH (3 days post operation) compared with that from sham‐operated mice. As shown in Figure 1A,B, the proportion of NK1.1^+^TCRb^−^ cells significantly decreased in 70% of PH mice. The immature CD27 SP NK cell population was significantly lower in the PH group than in the sham group. In contrast, the mature CD11b SP NK cell population increased significantly in the PH group (Figure 1A,C). As previously reported,^11^ the proportion of NK1.1^+^TCRb^−^DX5^−^ cells significantly decreased in 70% of PH mice (Figure 1A,D). Next, we analyzed NK cell receptor expression in each population of lr‐NK cells. Interestingly, immature CD27 SP NK cells, intermediate DP NK cells, and mature CD11b SP NK cells showed decreased expression of TRAIL and CD69 after PH (Figure 1E–H).

### 3.2 *AhR* contributes to the differentiation of TRAIL‐positive NK cells

**FIGURE 2:**
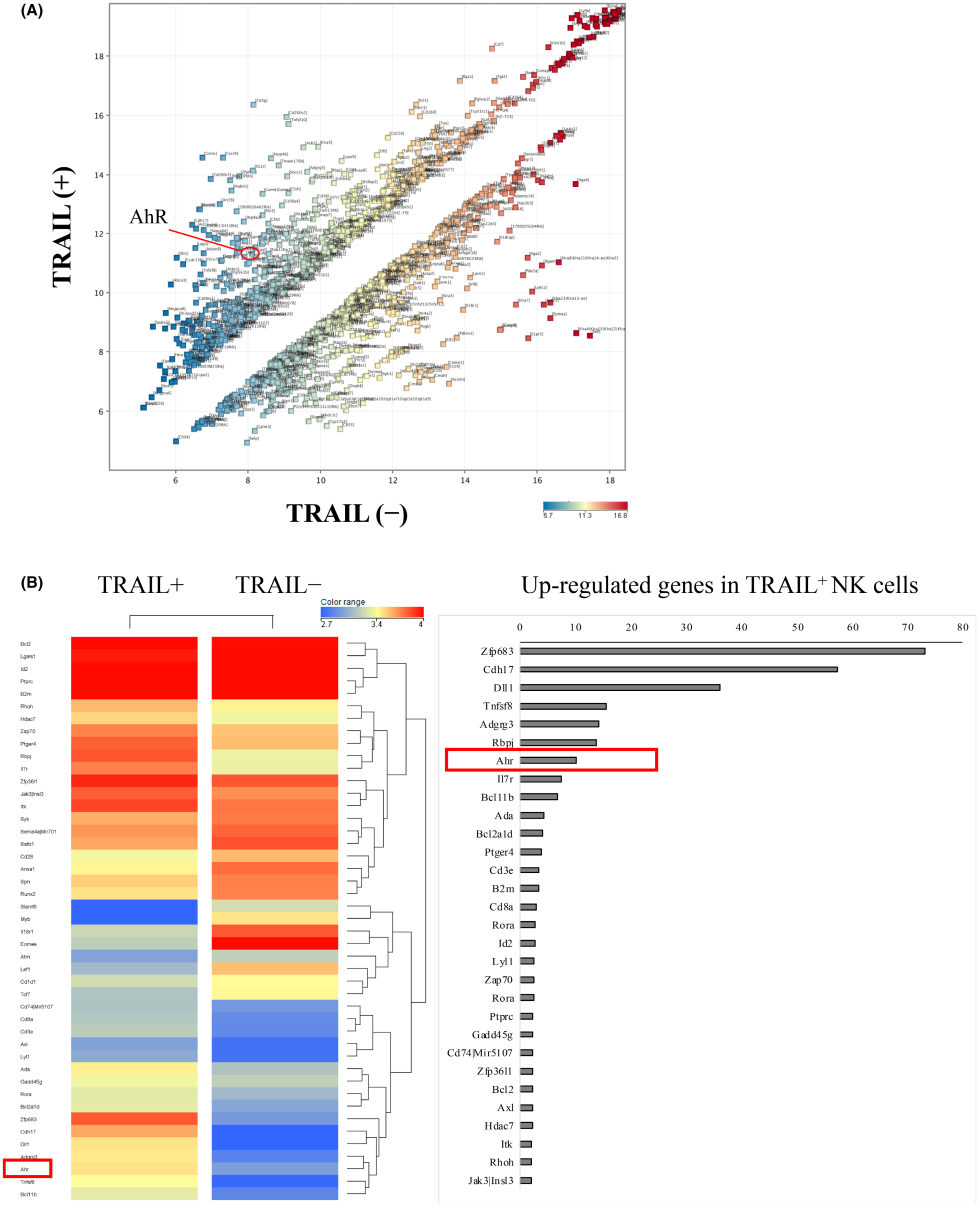
AhR was detected as a factor contributing to TRAIL expression on lr‐NK cells. (A) Total RNA from the TRAIL^−^ and TRAIL^+^ liver NK (NK1.1^+^TCRβ^−^) cells were characterized. The relative expression levels for each gene in the two types of NK cells were plotted against each other in the scatter plot. The gene‐level experiment combined the arithmetic mean of all probes mapping to the same probe ID. Fold‐change analysis was performed to eliminate probes that did not meet a 2.0‐foldchange. (B) 46 genes included in GO0030098 (lymphocyte differentiation) were selected; the heat map is shown.

Based on the observation that PH decreases the immature population of CD27 SP NK cells expressing TRAIL, we investigated the mechanisms that affect the differentiation of TRAIL‐expressing NK cells. To this end, we analyzed a microarray of liver‐resident NK cells to determine the genes involved in TRAIL expression. Gene expression microarray analysis was performed on sorted TRAIL^+^NK1.1^+^TCRβ^−^ and TRAIL^−^NK1.1^+^TCRβ^−^ lr‐NK cells. Among approximately 20,000 genes examined, we identified 1100 differentially expressed genes with >2‐fold change in expression level. Among these genes, 500 were overexpressed in the TRAIL^+^ NK cells (Figure 2A). Next, we performed Gene Ontology (GO) analysis to analyze the annotations existing in the genes strongly involved in TRAIL expression. GO is an annotation attached to a gene that focuses on its biological processes, cellular components, and molecular functions. When examining the GO terms attached to a gene, it is possible to estimate the function and intracellular localization of the gene. GO enrichment analysis was performed on 71 genes with a fold change >10 (upregulated genes) and GO terms with a *p*‐value <0.05. In total, 66 GO annotations were extracted (Table 1). Of these, we focused on GO0030098 (lymphocyte differentiation) because the development of NK cells changes after partial hepatectomy. There are 46 genes included in GO0030098, of which *AhR* has been reported to be related to NK maturation (Figure 2B). Based on the microarray data analysis of liver NK cells, we observed a significant upregulation in the mRNA expression of AhR (fold change = 10.1). This upregulation was associated with the expression of TRAIL in the NK cells, suggesting a dynamic regulation of AhR in relation to TRAIL expression in NK cells.

**TABLE 1:** GO enrichment analysis.

GO accession	GO term	GO accession	GO term
GO:0001910	Regulation of leukocyte mediated cytotoxicity	GO:0031343	Positive regulation of cell killing
GO:0001912	Positive regulation of leukocyte mediated cytotoxicity	GO:0032127	Dense core granule membrane
GO:0002376	Immune system process	GO:0034665	Integrin alpha1‐beta1 complex
GO:0002521	Leukocyte differentiation	GO:0040008	Regulation of growth
GO:0002682	Regulation of immune system process	GO:0044425	Membrane part
GO:0005102	Signaling receptor binding	GO:0044459	Plasma membrane part
GO:0005886|GO:0005904	Plasma membrane	GO:0046649	Lymphocyte activation
GO:0006955	Immune response	GO:0048534	Hematopoietic or lymphoid organ development
GO:0007154	Cell communication	GO:0048583	Regulation of response to stimulus
GO:0007165|GO:0023033	Signal transduction	GO:0048584	Positive regulation of response to stimulus
GO:0007166	Cell surface receptor signaling pathway	GO:0048585	Negative regulation of response to stimulus
GO:0008626	Granzyme‐mediated apoptotic signaling pathway	GO:0050896|GO:0051869	Response to stimulus
GO:0009109	Coenzyme catabolic process	GO:0050900	Leukocyte migration
GO:0009897	External side of plasma membrane	GO:0051239	Regulation of multicellular organismal process
GO:0009966|GO:0035466	Regulation of signal transduction	GO:0051241	Negative regulation of multicellular organismal process
GO:0009967|GO:0035468	Positive regulation of signal transduction	GO:0051270	Regulation of cellular component movement
GO:0009986|GO:0009928|GO:0009929	Cell surface	GO:0060844	Arterial endothelial cell fate commitment
GO:0010646	Regulation of cell communication	GO:0060846	Blood vessel endothelial cell fate commitment
GO:0010647	Positive regulation of cell communication	GO:0060847	Endothelial cell fate specification
GO:0016021	Integral component of membrane	GO:0060853	Notch signaling pathway involved in arterial endothelial cell fate commitment
GO:0018120	Peptidyl‐arginine ADP‐ribosylation	GO:0070481	Nuclear‐transcribed mRNA catabolic process, non‐stop decay
GO:0019221	Cytokine‐mediated signaling pathway	GO:0070887	Cellular response to chemical stimulus
GO:0019364	Pyridine nucleotide catabolic process	GO:0070966	Nuclear‐transcribed mRNA catabolic process, no‐go decay
GO:0019677|GO:0006737	NAD catabolic process	GO:0072526	Pyridine‐containing compound catabolic process
GO:0019835	Cytolysis	GO:0097101	Blood vessel endothelial cell fate specification
GO:0023051	Regulation of signaling	GO:0098552	Side of membrane
GO:0023052|GO:0023046|GO:0044700	signaling	GO:1901184	regulation of ERBB signaling pathway
GO:0023056	Positive regulation of signaling	GO:1902531|GO:0010627	Regulation of intracellular signal transduction
GO:0030097	Hemopoiesis	GO:2000026	Regulation of multicellular organismal development
GO:0030098|GO:0046650	Lymphocyte differentiation	GO:2000410	Regulation of thymocyte migration
GO:0030183|GO:0042115	B cell differentiation	GO:2000412	Positive regulation of thymocyte migration
GO:0030595	Leukocyte chemotaxis	GO:2001198	Regulation of dendritic cell differentiation
GO:0031224	Intrinsic component of membrane	GO:2001199	Negative regulation of dendritic cell differentiation

*Note*: GO enrichment analysis was performed on 71 genes with a fold change >10 (upregulated genes) and GO terms with a *p*‐value <0.05. A total of 66 GO annotations were extracted.

### 3.3 AhR agonists inhibit maturation and increase TRAIL expression and cytotoxicity in lr‐NK cells

**FIGURE 3:**
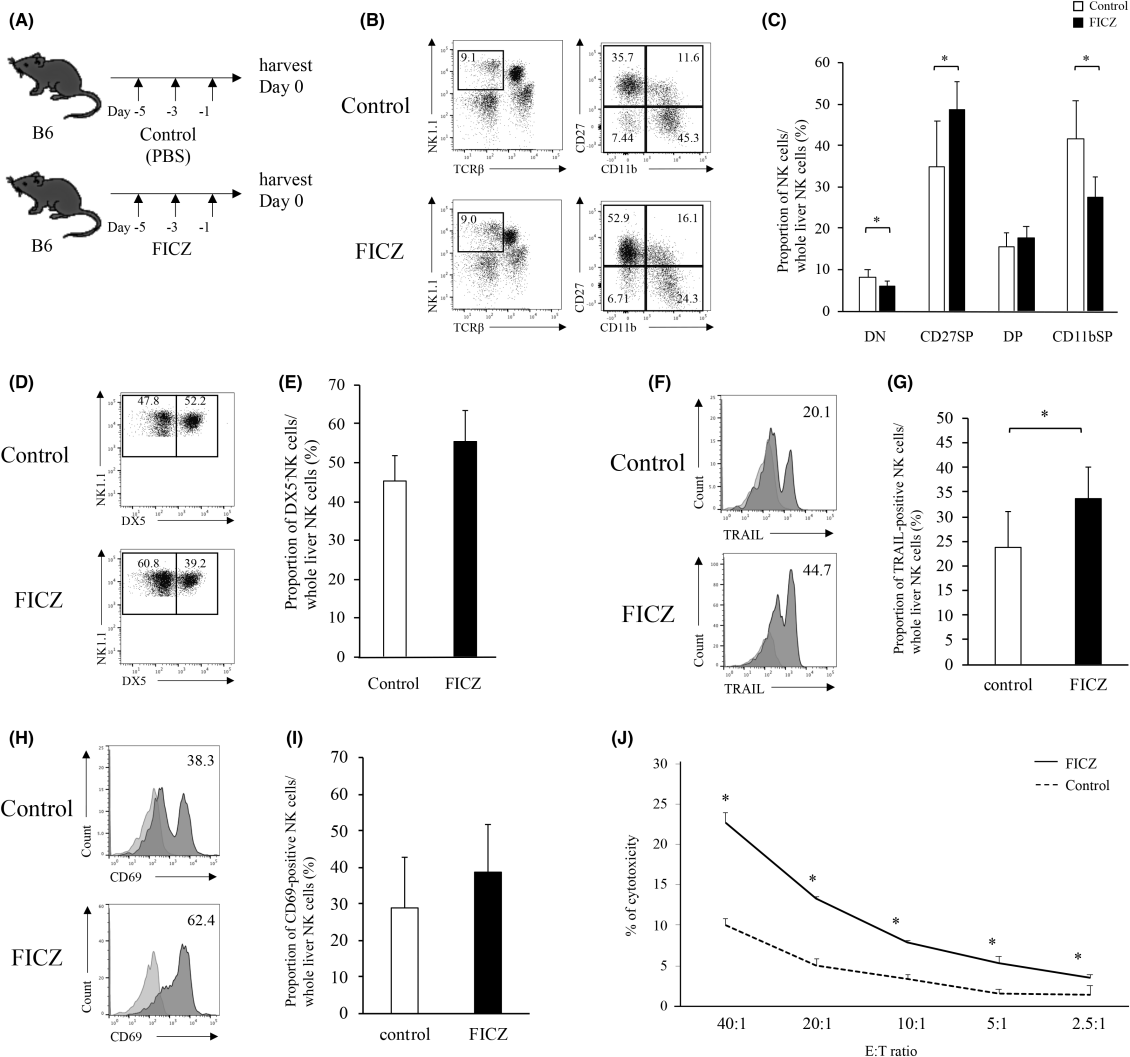
AhR agonist inhibits maturation and increases TRAIL expression and cytotoxicity in lr‐NK cells. (A) Schematic of FICZ injection. Data are expressed as mean ± SD. (B) Liver mononuclear cells were stained with anti‐NK1.1, anti‐TCRβ, and propidium iodide. NK1.1^+^TCRβ^−^ NK cells were then gated for the analysis of other markers (anti‐CD11b, anti‐CD27). Samples are shown in control (upper: *n* = 5) and FICZ‐treated mice (lower: *n* = 5). (C) Frequency of each lr‐NK cell subset in sham and FICZ‐treated mice. (D, E) Representative histograms for DX5^−^ expression are shown for control and FICZ‐treated mice. (F, G) Representative histograms for TRAIL expression in lr‐NK cells are shown for control (upper) and FICZ‐treated (lower) mice. The bar graph shows the average expression for TRAIL‐positive NK cells in control and FICZ‐treated mice. (H, I) Representative histograms for CD69 expression in lr‐NK cells are shown in control (upper) and FICZ‐treated (lower) mice. The bar graph shows the average expression of CD69‐positive NK cells in control and FICZ‐treated mice. (J) The cytotoxicity of lr‐NK cells is shown. Lr‐NK cells of FICZ‐treated mice had higher cytotoxicity compared to that observed with PBS. Data are expressed as mean ± SD (four mice per group). ANOVA was used to assess the statistical significance of differences between groups. *p < 0.05.

To examine the impact of AhR signaling on lymphopoiesis, the AhR agonist FICZ^14^ was injected into mice (Figure 3A). Serum levels of total bilirubin (T‐bil), aspartate transaminase (AST), and alanine aminotransferase (ALT), showed no significant differences between the control group and the FICZ‐treated group (Figure S1A–C). FICZ significantly increased the immature population of CD27 SP NK cells and decreased the mature population of CD11b SP NK cells in the liver (Figure 3B,C). FICZ administration tended to increase the proportion of DX5^−^ NK cells (Figure 3D,E); however, there were no statistically significant differences (*p* = 0.063) between the FICZ and control groups. TRAIL expression in NK cells significantly increased after FICZ administration, but no statistically significant increase in CD69 expression was observed (Figure 3F–I). Liver NK cytotoxicity against the hepatoma cell line was significantly increased after FICZ administration (Figure 3J). However, Liver NK cell cytotoxicity against CMT93 cells, a colon cancer cell line, was no difference between the control group and the FICZ‐treated group (Figure S2). Interestingly, administration of the AhR antagonist, 2‐methyl‐2H‐pyrazole‐3‐carboxylic acid (2‐methyl‐4‐*o*‐trilazo‐phenyl)‐amide, CH223191 which is a specific AhR inhibitor (Figure S3A),^19^ decreased the immature population of CD27 SP NK cells and increased the mature population of CD11b SP NK cells in the liver (Figure S3B,C). Concurrently, TRAIL and CD69 expression in NK cells significantly decreased after CH223191 administration (Figure S3B,D,E). Furthermore, the effect of CH223191 on lr‐NK cells was counteracted by the addition of FICZ (Figure S3B–E).

### 3.4 FICZ administration following partial hepatectomy can maintain attenuated TRAIL activity and tumor progression

**FIGURE 4:**
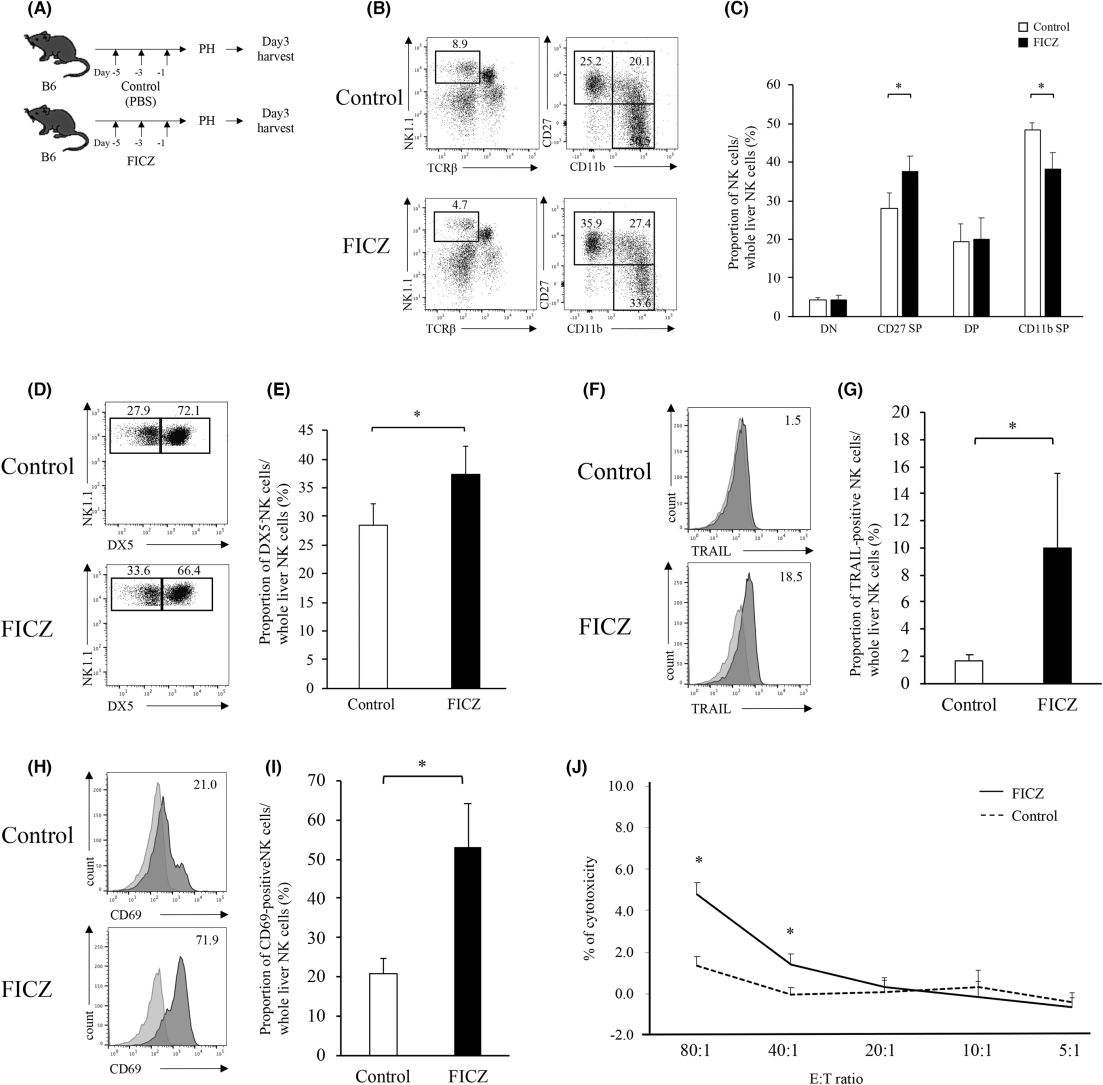
FICZ administration following partial hepatectomy can maintain attenuated TRAIL activity. (A) Schematic of FICZ or PBS as a control injection before PH (5 mice per group). (B, C) Liver mononuclear cells were stained with anti‐NK1.1, anti‐TCRβ, and propidium iodide. NK1.1^+^TCRβ^−^ NK cells were then gated for the analysis of other markers (anti‐CD11b, anti‐CD27). Specimens are shown for control (upper: *n* = 5) and FICZ‐treated mice (lower: *n* = 5). (C) Frequency of each subsets in lr‐NK cells in control or FICZ‐treated mice. (D, E) NK1.1^+^TCRβ^−^ NK cells were gated for the analysis of anti‐DX5. Representative histograms for DX5^−^ expression are shown for control and FICZ‐treated mice. (F, G) Representative histograms for TRAIL expression of lr‐NK cell are shown for control (upper) and FICZ‐treated (lower) mice. The bar graph shows the average expression for TRAIL‐positive NK cells in control or FICZ‐treated mice. (H, I) Representative histograms for CD69 expression of lr‐NK cells are shown for control (upper) and FICZ‐treated (lower) mice. The bar graph shows the average expression for CD69‐positive NK cells for control or FICZ‐treated mice. (J) The cytotoxicity of lr‐NK cells is shown. Lr‐NK cells of FICZ‐treated mice had higher cytotoxicity compared to that observed with PBS. Data are expressed as mean ± SD (four mice per group). Independent samples *t*‐tests were used to assess the statistical significance of differences between groups, when appropriate. *p < 0.05.

PH significantly decreases TRAIL expression in lr‐NK cells and NK cell cytotoxicity.^4^ Next, we examined whether FICZ could prevent the decrease in lr‐NK cell activity after PH (Figure 4A). Administration of FICZ before PH attenuated the decrease in immature CD27 SP NK cells and suppressed the increase in mature CD11b SP NK cells (Figure 4B,C). Administration of FICZ before PH markedly prevented the decrease in the proportion of DX5^−^ NK cells (37.3 ± 5.1 vs. 28.4 ± 3.9%; *p* = 0.024; Figure 4D,E), TRAIL expression (10.0 ± 5.5 vs. 1.6 ± 0.5%; *p* = 0.020), and CD69 expression (52.9 ± 11.3 vs. 21.0 ± 3.9%; *p* = 0.001) of lr‐NK cells compared with the vehicle control administration group on postoperative Day 3 (Figure 4F–I). FICZ administration before PH attenuated the decrease in liver NK cytotoxicity against the hepatoma cell line (Figure 4J).

**FIGURE 5:**
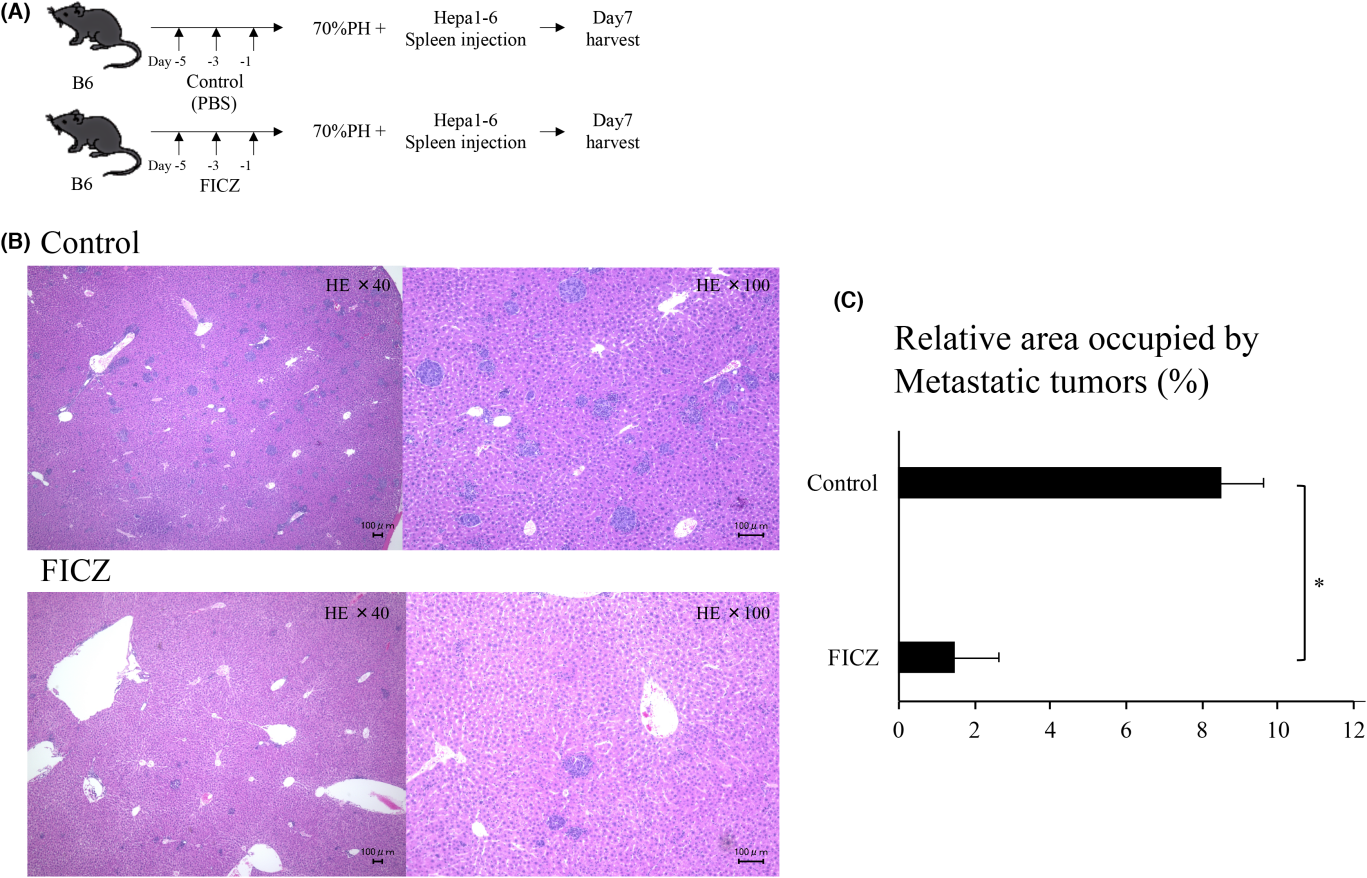
FICZ administration attenuated tumor progression. (A) Schematic representation of FICZ injection in liver metastasis model. (B) Representative histopathological findings of liver specimens after tumor injection (stained with hematoxylin and eosin stain). The scale bar in small micrographs represents 100 μm. Samples are shown from PBS‐treated PH (upper) and FICZ‐treated PH mice (below; four mice per group). Independent samples *t*‐tests were used to assess the statistical significance of differences between groups, when appropriate. *p < 0.05.

To assess the impact of FICZ administration on metastatic growth in the liver, Hepa1‐6 cells (2 × 10^6^) were injected into the spleen of the mice that underwent PH, either treated with FICZ or receiving a vehicle control (PBS). After 7 days, the mice were sacrificed, and the presence of metastatic lesions in the liver was evaluated (Figure 5A). In FICZ‐treated mice, a reduced number of metastatic lesions were observed compared to the vehicle control group, indicating their sustained innate defensive activity against invading hepatoma cells upon FICZ administration (Figure 5B,C).

### 3.5 AhR regulates the expression of TRAIL molecules through differentiation‐related signals

**FIGURE 6:**
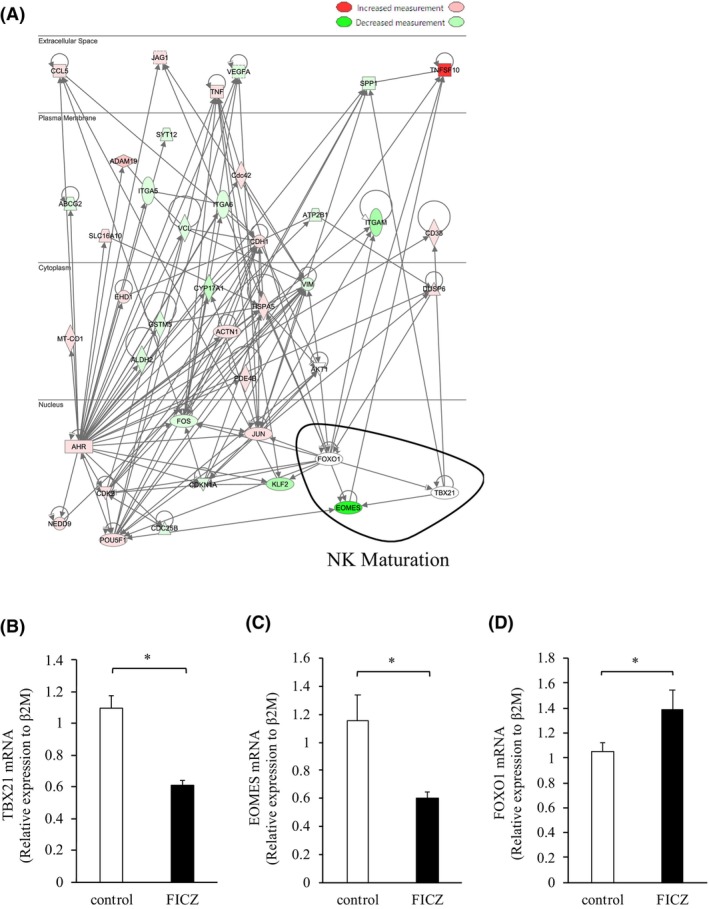
AhR regulates the expression of TRAIL molecules through differentiation‐related signals. (A) Ingenuity pathway analysis was used to predict upstream regulators that are differentially activated between AhR and TNFSF10. (B–D) Forkhead box O (FoxO), Tbx‐21 (T‐bet), and Eomesodermin (Eomes) in lr‐NK cells were analyzed using quantitative real‐time polymerase chain reaction (PCR) after treatment with PBS (control) or FICZ (four mice per group). Independent samples *t*‐tests were used to assess the statistical significance of differences between groups, when appropriate. *p < 0.05.

**FIGURE 7:**
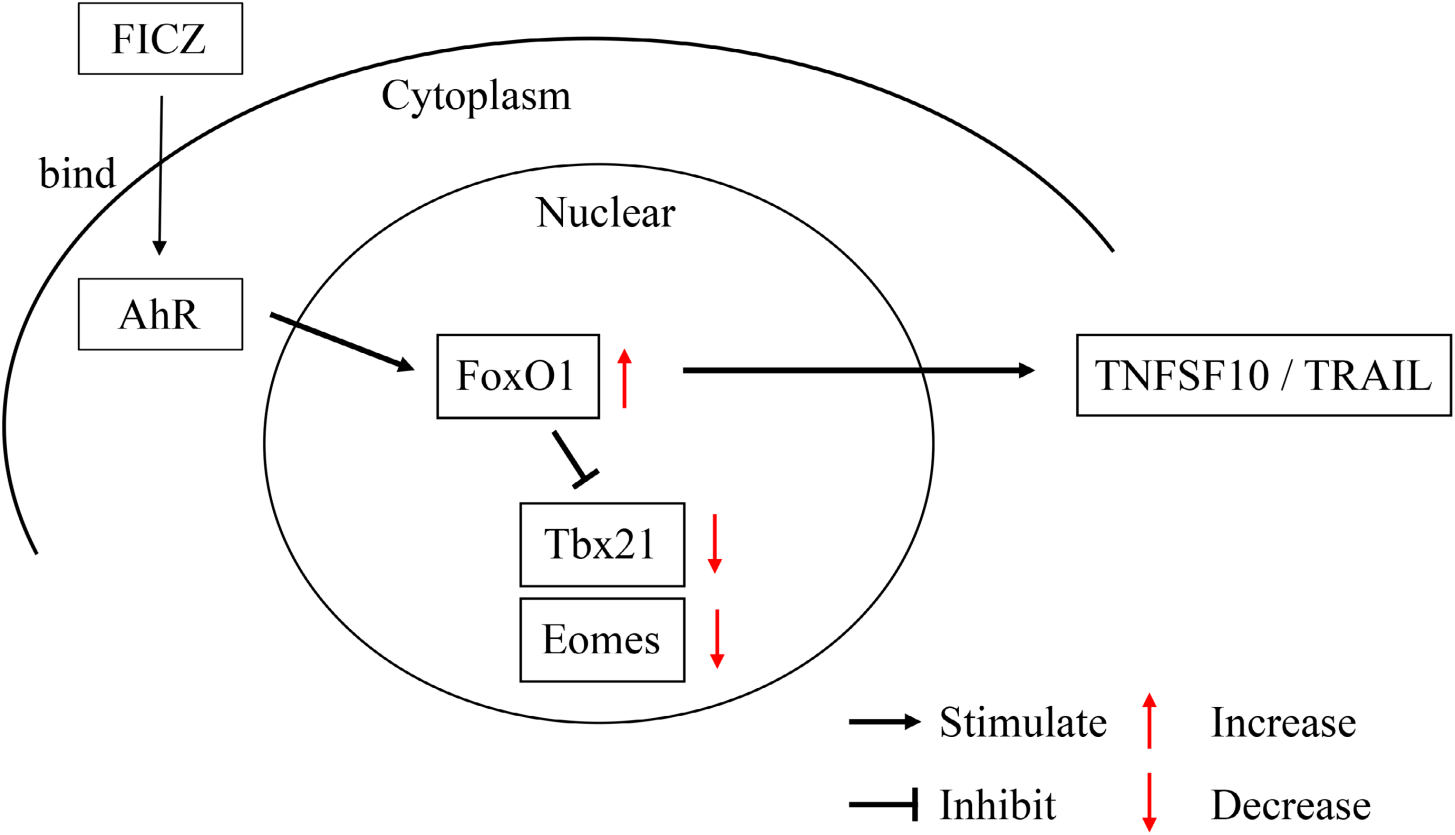
Summary of FICZ‐AhR signaling in lr‐NK cells. FoxO1 regulates NK cell development through T‐bet. FoxO, T‐bet, and Eomes in lr‐NK cells are presented according to the results of quantitative real‐time PCR. FoxO, Forkhead box O. T‐bet, Tbx‐21. Eomes, Eomesodermin. TRAIL, TNF‐related apoptosis‐inducing ligand. TNFSF10, TNF superfamily member 10.

To analyze the involvement of AhR in TRAIL expression in NK cells, we performed Ingenuity Pathway Analysis (IPA) to predict the upstream regulators that were differentially activated between AhR and TNFSF10 (Figure 6A). Here, the AhR to TNFSF10 signaling pathways in lr‐NK cells were analyzed using qRT‐PCR after FICZ administration. Consistent with FICZ stimulation in lr‐NK cells, the mRNA expression of Tbx‐21 (T‐bet) and Eomes in lr‐NK cells decreased significantly after FICZ administration. In contrast, FoxO1 mRNA expression in lr‐NK cells increased significantly after FICZ administration (*p* < 0.05; Figure 6B–D). The transcription factors T‐bet and Eomes play a role in NK cell development.^6^, ^20^ FoxO1 is a negative regulator of NK cell differentiation and function. Immature NK cells exhibit high levels of FoxO1 expression and low levels of Tbx21 compared to mature NK cells. Interestingly, as NK cell development progresses, the expression patterns of these two transcription factors show opposite trends.^21^ The IPA of lr‐NK cells is summarized in Figure 7.

## 4 DISCUSSION

In this study, we elucidated the mechanism by which PH decreases lr‐NK cell activity and identified AhR as a potential therapeutic agent. AhR–FICZ signaling increased the proportion of TRAIL^+^ NK cells and the cytotoxicity of lr‐NK cells. Administration of FICZ before PH canceled this attenuation. Our experiments revealed that lr‐NK cell expression of the maturation gene is altered by FICZ–AhR signaling and involved in TRAIL expression. First, we showed that PH alters the maturation of lr‐NK cells. The number of the immature subtype of NK cells (CD27 SP NK) expressing high TRAIL activity decreased after PH; simultaneously, that of the mature type of NK cells (CD11b SP NK) expressing low TRAIL activity increased after PH. Second, through microarray studies, we found that AhR contributed to the differentiation of TRAIL^+^ NK cells. Third, we showed that the AhR agonist, FICZ, inhibited the maturation of and increased TRAIL expression and cytotoxicity of lr‐NK cells. Furthermore, FICZ administration after PH maintained attenuated TRAIL activity and tumor progression. Finally, we found that AhR regulates the expression of TRAIL molecules through differentiation‐related signals. Taken together, our data suggest that PH decreases lr‐NK cell activity via the AhR pathway.

We have previously reported that TRAIL expression in lr‐NK cells decreased markedly after PH^4^; however, the underlying mechanism remains unknown. In this study, the substantial reduction in the population of TRAIL^+^ NK cells in the liver after PH could be explained by the maturation of NK cells. The relationship between maturation changes in NK cells and their TRAIL expression is consistent with the results of our previous study.^10^ There are few reports on the phenotypic changes in NK cells after PH. An opposite result was observed for the phenotypic changes of NK cells after PH: there was a loss of the mature CD11b SP NK cell subset after PH.^22^ The PH model used in this previous study was a lethal model that ligated approximately 90% (median, left, and right inferior lobes) of the liver. As such a model cannot be used as a clinical model, it is possible that the opposite result was obtained. However, our model was a clinical one that can survive for a long time. Our model is closer to the clinical scenario and provides valuable insights into the phenotypic changes of NK cells following PH. Our findings suggest that changes in the lr‐NK cell maturation are responsible for the reduced TRAIL expression after PH.

In this study, microarray and GO analyses performed to search for genes involved in TRAIL expression and cell differentiation in NK cells revealed that *AhR* is dynamically upregulated in TRAIL^+^ NK cells and strongly involved in the differentiation of NK cells. The immunoregulatory effects of AhR exhibit variations across different immune cell types. Many studies have explored the multifaceted role of AhR signaling and effectors in dendritic cells (DCs), T cells, and regulatory T cells differentiation.^23^, ^24^ However, limited research studies have focused on the relationship between AhR and NK cells. In some of them, AhR has been previously implicated in the development, maintenance, and function of NK cells.^14^ Furthermore, few studies have investigated the association between AhR and NK cell maturation. Freund et al. reported that the expression of AhR in NK cells from human secondary lymphoid tissues decreases during NK cell maturation,^25^ and Moreno‐Nieves et al. found that in humans, AhR expression was higher in immature peripheral NK cells compared to the mature cells.^26^

We used FICZ as an AhR agonist to enhance TRAIL activity in NK cells. FICZ is a TRP‐derived AhR ligand that binds to AhR with the highest affinity.^27^ Our results showed that the administration of AhR agonists altered the maturation and TRAIL expression of NK cells. In other words, administration of the AhR agonist maintained NK cells in their immature form and prevented their development into the mature type, resulting in an increase in TRAIL activity. Furthermore, the AhR agonist prevented the decrease in TRAIL activity in NK cells, even after PH. These findings suggest that AhR activation through agonist administration can modulate the maturation and TRAIL expression of lr‐NK cells, potentially enhancing their immune functions. Our study focused on the AhR agonists affecting lr‐NK cell maturation and TRAIL expression; few studies have analyzed the antitumor activity of NK cells with FICZ in an in vivo mouse model. Shin et al. demonstrated that activating AhR with FICZ can enhance the IFN‐γ secretion by NK cells and simultaneously increase their antitumor activity against RMA‐S lymphoma tumors in an NK cell‐dependent manner.^16^ In this study, FICZ administration inhibited liver metastasis in an in vivo PH mouse model. This may mitigate reduced host defense following hepatectomy or liver transplantation (LT). Tumor recurrence is the main limitation of hepatectomy and LT in patients with hepatocellular carcinoma (HCC).

We have previously reported an adoptive immunotherapy approach that uses lr‐NK cells derived from a donor liver graft perfusate to prevent tumor recurrence after LT.^28^ FICZ may be an additional option in adaptive immunotherapy.

Recently, combination therapies of immune checkpoint inhibitors (ICIs) with multikinase inhibitors have shown superior clinical efficacy.^29^ The most studied ICIs currently target programmed cell death‐1 (PD‐1), its ligand PD‐L1, and cytotoxic T lymphocyte‐associated protein 4, with proven efficacy in advanced HCC.^30^ Indoleamine 2,3‐dioxygenase (IDO) is overexpressed in HCC; it is a kynurenine pathway enzyme responsible for degrading tryptophan, an AhR agonist. The inhibition of IDO in combination with ICIs may enhance therapeutic efficacy.^31^ IDO is induced by innate immune responses during tumorigenesis and by attempts to activate T cells, either spontaneously or via immunotherapy.^32^ A phase III study of IDO inhibitors with ICIs in unresectable melanoma has been reported; however, IDO inhibitors plus PD‐1 inhibitors do not improve patient outcomes relative to the placebo plus PD‐1 inhibitors.^33^ Potential drug interactions of IDO inhibitors with ICI combination therapies require further investigation. Furthermore, in patients with cirrhosis infected by chronic viral diseases, such as hepatitis C virus (HCV) infection, antiviral therapy contributes in preventing HCC recurrence after liver resection.^34^ NK cells have been implicated in protective immune responses against hepatitis B virus (HBV) and HCV infection and the suppression of liver fibrosis and HCC.^35^ We have previously reported that HCV infection is completely prevented in human hepatocyte chimeric mice by injecting activated human liver‐derived lymphocytes after inoculation with HCV‐infected human serum.^36^ Adaptive immunotherapy using enhanced NK activity may also contribute to effective antiviral therapy in patients with HCC manifesting underlying chronic viral liver disease.

In this study, we investigated the signaling pathways using IPA between AhR and TNFSF10 in NK cell maturation. By analyzing gene expression, we identified FoxO1, T‐bet, and Eomes as key regulators involved in this process. Gordon et al. indicated the significance of T‐bet in determining the developmental stability of immature NK cells expressing TRAIL. T‐bet suppresses CD27 expression, thereby promoting the transition from a CD27‐positive immature state to a CD27‐negative mature state of NK cells.^6^ Additionally, the transcription factor Eomes is necessary for the maturation process and characterizes the reduction of TRAIL expression and induction of integrin DX5 expression.^6^ Furthermore, FoxO1 represses T‐bet expression via direct binding to the T‐bet promoter and serves as a negative checkpoint in NK cell maturation.^21^

In our study, FICZ treatment resulted in the increased expression of FoxO1 and decreased expressions of T‐bet and Eomes in lr‐NK cells. These findings are consistent with our previous results, which showed an expanded population of immature lr‐NK cells and higher TRAIL expression associated with elevated FoxO1 levels and reduced T‐bet and Eomes expressions.^10^ These results suggest that FICZ activation influences the expression of key transcription factors involved in NK cell maturation and impacts the TRAIL expression of lr‐NK cells via AhR signaling.

AhR has been suggested to be a promoter of tumor cell development and progression, but this view is controversial.^37^, ^38^ Several investigations have provided evidence supporting the potential tumor‐suppressive role of AhR under certain circumstances.^39^ Fan et al. suggested that AhR activation by ITE was significantly efficacious in suppressing tumor growth in HCC cells, primarily by inducing cell cycle arrest at the G1/G0 phase, promoting apoptosis, and inhibiting the migration and invasion processes.^39^ Kawajiri et al. reported that AhR has a role in suppressing intestinal carcinogenesis through the AhR ligand‐dependent β‐catenin degradation pathway.^40^ Furthermore, recent studies have demonstrated compelling data to support the vital role of AhR signaling in innate immunity.^14^, ^41^, ^42^ In addition, regeneration of the liver after PH improves in the absence of AhR.^43^ The transient inhibition of AhR may improve liver regeneration in patients with liver resection and transplant. In this study, AhR had been involved in cell proliferation and the induction of inflammatory cytokines, tumor necrosis factor‐alpha (TNF‐α), and interleukin (IL)‐6 in the early phase after PH.^43^ Liver regeneration after PH is regulated by various immune cells through direct interaction with hepatocytes or indirectly by releasing inflammatory cytokines.^44^ NK and NKT cells have a high potential to upregulate TNF‐α and IL‐6 and may promote normal regenerative responses in the liver.^45^ The involvement of lr‐NK cells via AhR in liver regeneration and their antitumor effects on intrahepatic immunity after PH require further research. Exploration of these clinical treatments may be worth pursuing for pharmaceutical scientists.

However, this study had some limitations. First, the roles of other immune cells expressing AhR, such as regulatory T cells, CD4^+^/CD8^+^ T cells, and DCs, were not evaluated. AhR plays an important role in regulating the differentiation between Treg cells and Th17 cells, which are pivotal in autoimmune diseases.^46^ FICZ stimulates the differentiation and maturation of dendritic cells (DCs), as well as induces the differentiation of naïve T cells into Foxp3^+^ Treg‐like cells. These effects of FICZ contribute to the induction of immune tolerance.^47^ Second, our study indicates a link between AHR and FoxO1; however, this link is based largely on pathway analysis. Previous studies indicated that FoxO1 regulates NK cell development through T‐bet,^21^ and NK cell maturity is positively correlated with T‐bet expression but negatively correlated with AhR expression.^25^ These results support our findings that AhR signaling increases immature subtypes of NK cells, and increases FoxO1 expression while decreasing T‐bet expression. Although these results show a negative correlation between the expression of AhR and T‐bet regulated by FoxO1 through NK cell development, they do not indicate that FoxO1 is regulated directly or indirectly via AhR signaling. Future studies are warranted to determine whether FoxO1 is regulated directly or indirectly by AHR, and the change in NK cell developmental intermediates is due to a shift in maturation, apoptosis of selective proportions, or other causes. Third, although our results indicate a direct mechanism for AhR‐mediated activity of lr‐NK cells, the results show that an additional AhR signal can maintain lr‐NK cell activity. Other pathways and an indirect mechanism may be responsible for suppressing the activity of lr‐NK cells, and this should be investigated in future studies. Fourth, in this study, we evaluated the post‐PH activity of lr‐NK in the normal liver of B6 mice but not in different liver backgrounds such as non‐alcoholic fatty liver disease (NAFLD) and non‐alcoholic steatohepatitis (NASH). To date, the regulation and function of lr‐NK cells in NAFLD and NASH remain controversial.^48^ Stiglund et al. have reported that the phenotype and frequency of liver NK cells are unaltered in patients with NAFLD and NASH.^49^ Kahraman et al. have reported that patients with NALFD have decreased numbers of liver NK cells, while those with NASH exhibit high NKG2D expression in the liver parenchyma, which is associated with increased liver NK cells with elevated gene and protein expression of its ligand, major histocompatibility complex class I–related chains A/B.^50^ Tosello‐Trampont et al. have reported that in a mouse model of NASH, TRAIL expression decreases, IFN‐γ production increases, and cytotoxicity is unaltered during NASH progression in liver NK cells.^51^ Further studies are needed to evaluate whether AhR signaling can maintain attenuated TRAIL activity and tumor progression after PH even if TRAIL activity and cytotoxicity of lr‐NK cells are reduced in NAFLD and NASH models. Moreover, NK cells are involved in protective immune responses against HBV and HCV infection as well as in the suppression of liver fibrosis and HCC.^35^ Liver NK cells play a major role in the removal of activated hepatic stellate cells or liver cancerous cells from fibrotic livers via TRAIL and/or NKG2D signaling.^52^, ^53^ Many immune cells, including NK cells, become dysregulated as the disease develops from liver cirrhosis to HCC.^54^ The occurrence of HCC is well‐known among patients with liver cirrhosis. In advanced cirrhosis, the feasibility of surgical resection may reduce. The effect of cirrhosis‐induced changes on the functions of lr‐NK cells needs to be evaluated, to further identify new therapeutic targets and design immunotherapeutic strategies.

## 5 CONCLUSION

We found that AhR signaling using FICZ administration could maintain TRAIL expression in lr‐NK cells, even after PH. Our findings indicate that the modulation of T‐bet and Eomes affects TRAIL expression in lr‐NK cells via AhR in mice. These results have important implications for the future treatment of hepatocellular carcinoma. Perioperative therapies using an AhR agonist to improve NK cell function may reduce the recurrence of hepatocellular carcinoma after hepatectomy.

## AUTHOR CONTRIBUTIONS

**Koki Sato:** Investigation (lead); writing – original draft (lead). **Masahiro Ohira:** Conceptualization (equal); supervision (equal). **Yuki Imaoka:** Investigation (equal); resources (equal). **Kouki Imaoka:** Investigation (supporting); validation (equal). **Tomoaki Bekki:** Investigation (supporting); resources (supporting). **Marlen Doskali:** Investigation (supporting); resources (supporting). **Ryosuke Nakano:** Investigation (supporting); supervision (supporting). **Takuya Yano:** Methodology (supporting); resources (supporting). **Yuka Tanaka:** Conceptualization (supporting); supervision (supporting). **Hideki Ohdan:** Conceptualization (lead); supervision (lead).

## FUNDING INFORMATION

This work was supported in part by JSPS KAKENHI, grant numbers JP23H02981, JP22H00479, JP22K16534 and JP22K16535; and AMED, grant numbers JP23fk0210108.

## CONFLICT OF INTEREST STATEMENT

The authors declare that the research was conducted in the absence of any commercial or financial relationships that could be construed as a potential conflict of interest.

## ETHICS STATEMENT

The study was carried out strictly in accordance with the Guide for the Care and Use of Laboratory Animals and the local committee for animal experiments. The experimental protocol was approved by the Ethics Review Committee for Animal Experimentation of the Graduate School of Biomedical Sciences, Hiroshima University (approval number: A20‐97).

## Supporting information


Figures S1–S3.

Table S1:

## Data Availability

Data sharing is not applicable to this article as no new data were created or analyzed in this study.
