# Supplementary material for: The aryl hydrocarbon receptor maintains antitumor activity of liver resident natural killer cells after partial hepatectomy in C57BL/6J mice
Source: Cancer Med. 2023 Sep 25;12(19):19821–37. doi: 10.1002/cam4.6554 (PMC10587932; doi:10.1002/cam4.6554)
Supplement: Supplementary file 1 — Figures S1–S3. [file CAM4-12-19821-s002.zip › cam46554-sup-0001-Figures1-s3/Figure S1.docx]

Figure S1. Serum levels of T-bil/AST/ALT in FICZ or PBS- (Control) treated mice. Mice received three intravenous injections of 3 mg/300 μL of FICZ or 300 μL of PBS for 1 week (four mice per group).

Figure S2: Liver NK cell cytotoxicity against CMT93 cells, a colon cancer cell line, in FICZ or PBS- (Control) treated mice. There was no difference in the liver NK cell cytotoxicity against the colon cancer cell line between the control group and the FICZ-treated group.

Figure S3: Aryl hydrocarbon receptor (AhR) antagonist promotes maturation and decreases TRAIL expression in liver-resident natural killer (lr-NK) cells. Mice received three intraperitoneal injections of 1 mg/kg of CH223191 or 300 μL of phosphate-buffered saline (PBS) or injections of 3 mg/300 μL of FICZ plus CH223191 for 1 week (four mice per group). (A) Schematic representation of the dosing regimen. Data are expressed as mean ± standard div SD. (B) Samples are shown in control (upper), CH223191-treated mice (middle), and CH223191plus FICZ-treated mice (lower). Liver mononuclear cells were stained with anti-NK1.1, anti-TCRβ, and propidium iodide. NK1.1^+^TCRβ^−^ NK cells were then gated for the analysis of other markers (anti-CD11b, anti-CD27, TRAIL, CD69). (C) Proportion of lr-NK cell subsets in control and CH223191 and CH223191 plus FICZ-treated mice. (D) The bar graph shows the average expression of TRAIL-positive NK cells for the control, CH223191, and CH223191 plus FICZ-treated mice. (E) The bar graph shows the average expression of CD69-positive NK cells in the control, CH223191, and CH223191 plus FICZ-treated mice. Independent samples t-tests were used to assess the statistical significance of differences between groups, when appropriate. **p* < 0.05.
