# Supplementary figures and images for: The aryl hydrocarbon receptor maintains antitumor activity of liver resident natural killer cells after partial hepatectomy in C57BL/6J mice
Source: Cancer Med. 2023 Sep 25;12(19):19821–37. doi: 10.1002/cam4.6554 (PMC10587932; doi:10.1002/cam4.6554)

Supplementary Figure. 1

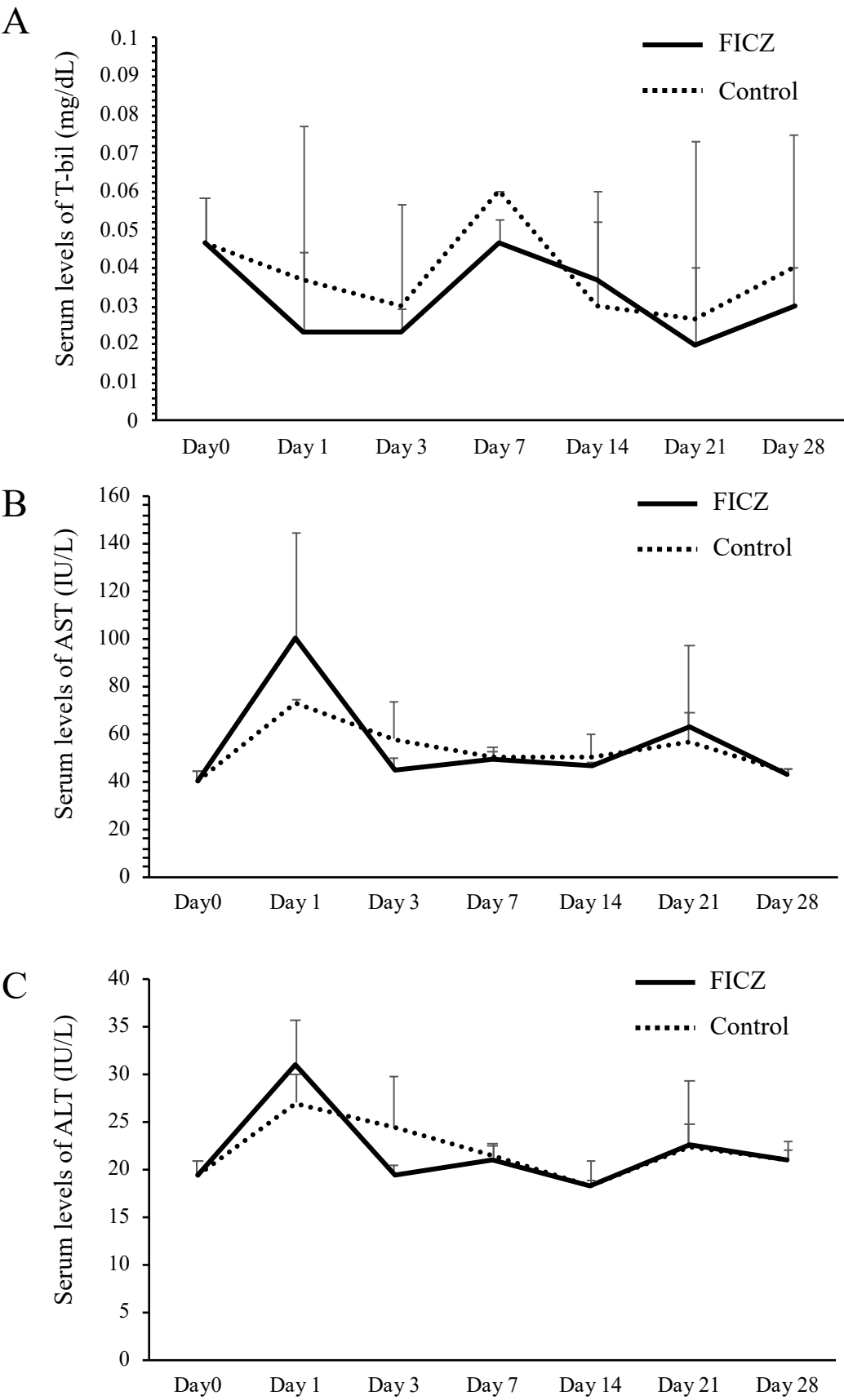

Supplementary Figure. 2

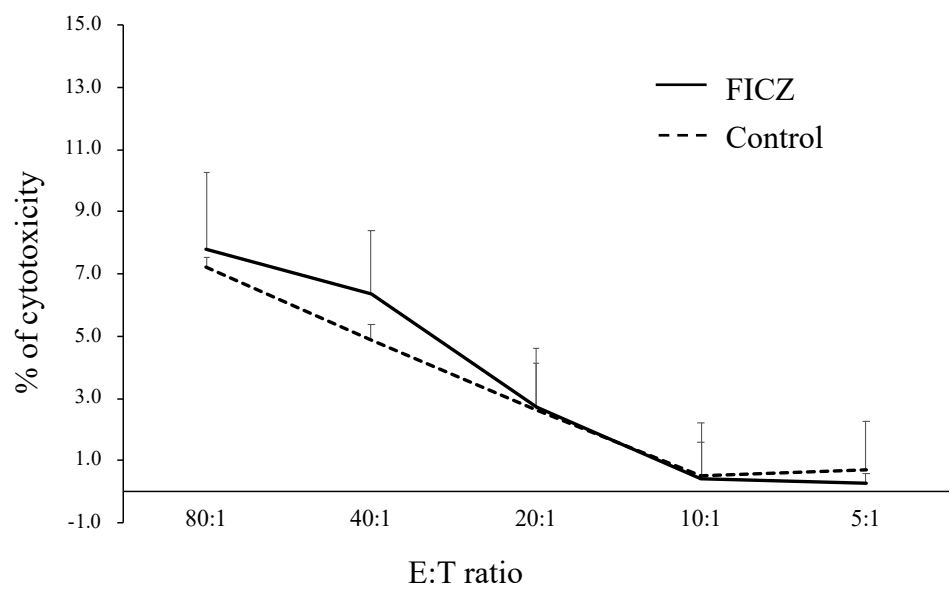

Supplementary Figure. 3

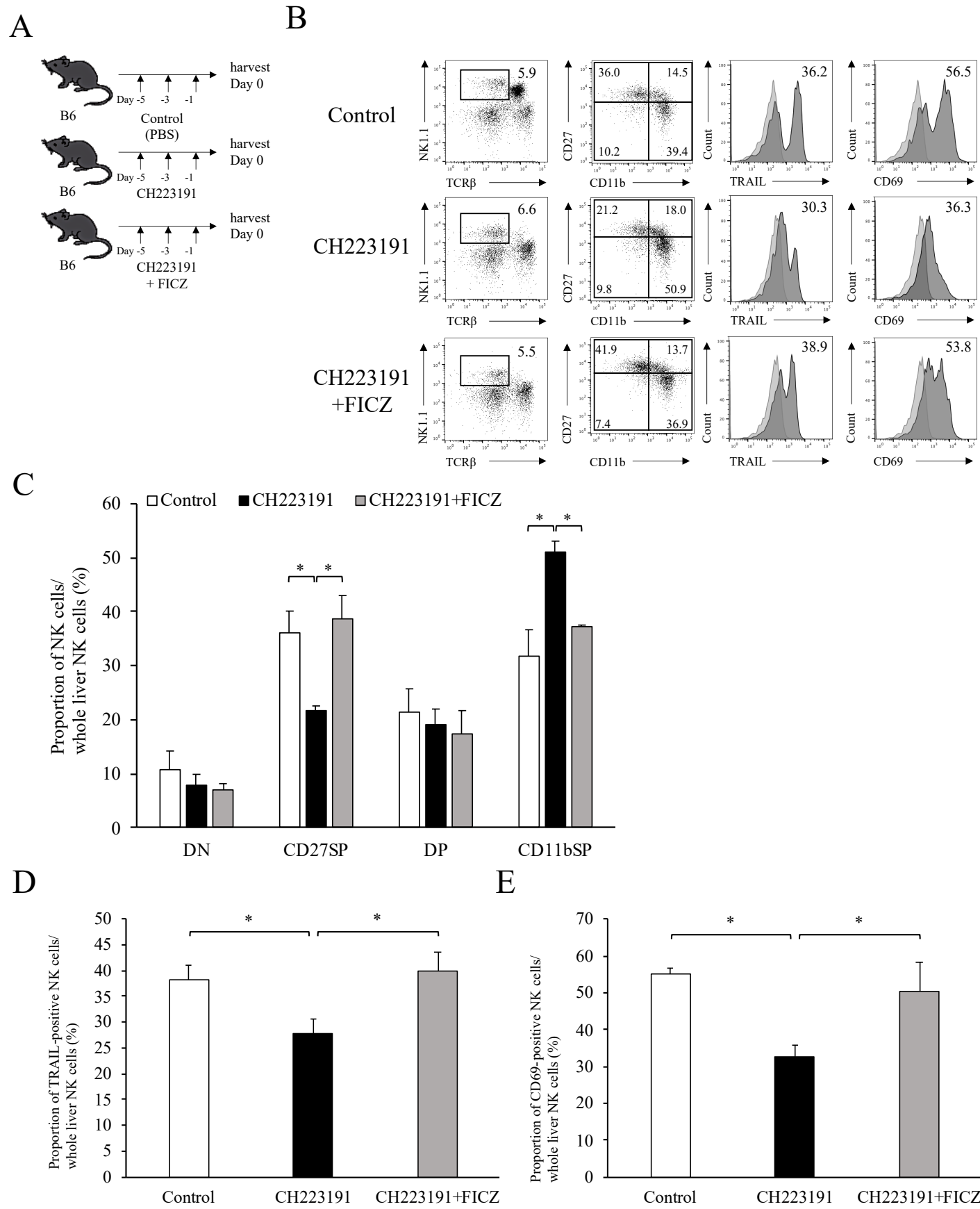

Supplement: Supplementary file 1 — Figures S1–S3. [file CAM4-12-19821-s002.zip › cam46554-sup-0001-Figures1-s3/cam46554-sup-0001-Figures.pdf]
